# Management of the Inlay Matrix

**Published:** 1904-06-15

**Authors:** L. E. Custer

**Affiliations:** Dayton, O.


### MANAGEMENT OF THE INLAY MATRIX.

BY L. E. CUSTER, A.M., D.D.S., DAYTON, O.

Read before the Ohio State Dental Society, December, 1903, and printed from advance proofs by courtesy of "Dental Summary."

One of the first problems that the beginner in porcelain inlay work meets is the tendency of the matrix to change shape under the contracting influence of the porcelain. All porcelains lose bulk in passing from the powder to the solid form. This takes place for reasons well known. The amount of shrinkage in bulk under proper fusing depends largely upon the condition of the powder before fusing. The powder being formed into a paste by the addition of water and placed into the matrix, if lightly jarred will settle and become fairly compact. The condensation can be further carried on by absorbing the water in a napkin, adding fresh water and again jarring and absorbing the moisture. This can be repeated several times to advantage. The jarring is best done by drawing a knurled instrument across the tweezers with which the matrix is held. The thorough condensation of the porcelain prior to fusing not only reduces the amount of shrinkage but insures an inlay of fine texture and much time should therefore be given to this step. However, in spite of all the care that one can give to the act of condensation, there will always be a shrinkage in bulk when the powder has become a solid mass and this shrinkage will always be a problem in inlay work. It is a property of porcelain when fused upon a piece of metal to draw the metal to it as it passes into the solid state. If, however, the metal is sufficiently rigid, then the porcelain appears to melt down to the metal without changing its shape. This is demonstrated in every case of continuous gum, and it is surprising how thin the plate may be and yet control the porcelain when passing from the liquid to the solid state. In inlay work, however, it is necessary to use metal exceedingly thin, so thin that it has little natural stiffness to resist the draw of the porcelain. Herein lies the most important problem in inlay work; when the matrix is thin enough to serve its purpose it has not sufficient stiffness to control the porcelain. The tendency of the matrix to change shape increases with the size of the cavity; first, because there is greater loss of bulk in the porcelain, and, second, because a large matrix is more easily bent than a small one. A long strip of metal will bend by its own weight.

The first step then would be one directed to the strengthening of the matrix itself by giving it such a form as to stiffen it against the contractive influence of the porcelain. It is desirable to use the matrix as thin as is consistent with proper handling in order not only to secure a more perfect adaptation to the cavity walls themselves but also to reduce the discrepancy of fit due to the thickness of the matrix. Except in those cavities whose margins assume a bell shape and where a little is dressed from the bottom of the inlay there will always be an incomplete adaptation at the margins proportionate to the thickness of the matrix material. It, therefore, being desirable to use the matrix very thin it is possible to stiffen it against the contractive influence of the porcelain by turning the margins of the matrix at right angles to the cavity. If a wide margin is left to the matrix and this is turned over the edge of the cavity so as to form an unbroken rim it not only defines the cavity outlined but stiffens the matrix at the cavity margin to an extent that is usually sufficient to resist a change of shape. This course is practical in all crown cavities of bicuspids and molars and in the labial cavities of the anterior teeth. In approximal cavities it can only be carried out where there is sufficient space and where the cavity does not extend too far cervically. Even where this can not be done at the cervix, it should be carried out at all other margins and the cervix treated in a manner to be described.

The use of small rods of high-fusing porcelain dropped into the matrix and the new porcelain flowed around the same was first suggested by Dr. J. W. Wassall. These rods not only lessen the amount of shrinkable new porcelain but act as supports to the matrix walls. They are now made by Mr. Brewster and can also be made by the dentist if he will fuse a strip of high-fusing porcelain and cut up the same with wedge-cutters. In using these rods, one, two or three as the cavity permits are selected of such a length that when dropped into the matrix they will lodge upon the sides before reaching the bottom. The porcelain is then flowed around them and when fused it will be found that the matrix has been supported so well that the porcelain has contracted toward the rods and matrix and a change of shape has been prevented.

The third procedure, and one to which I would call special attention is the use of a thin layer of comparatively high-fusing porcelain upon the matrix. The Brewster outfit contains what is termed "foundation body," which was primarily intended as a foundation for bulk and color, but this when followed by the enamels of the same outfit does not possess sufficient difference in the fusing point for the purpose indicated. Experiments were made with higher-fusing bodies and it was found that a mixture of Close, four parts, and Beauman body, one part, made a body of such high-fusing point that it remained unchanged when the Brewster enamel was fused thereon. After further experiments it was found that the Beauman body might be left out entirely provided the Brewster enamel was not at any time overfused.

In making an inlay by this method, I proceed as follows: A fairly good adaptation of the matrix to the cavity is obtained. This is then covered with a very thin layer of Close body, which naturally flows deeper at the bottom than upon the sides. After being fused it is returned to the cavity and at this stage much care is given to the adaptation of the matrix to the walls of the cavity. A large pellet of cotton is placed in the matrix and compressed with considerable force equally distributed. Upon removing the cotton it will be found that the matrix which before would rock in the cavity is now firmly seated. The porcelain is cracked in many places but still adherent to the matrix. Care is now taken that all the margins are carefully adapted not by prolonged burnishing but by delicately pressing the metal against the cavity margin. After the matrix has gone through the heat of the first fusing it will be found to be extremely dead. The moment a burnisher is rubbed across it, it is endowed with temper and spring. With one instrument I steady the matrix while with another I go entirely around the margins carefully and delicately pressing the metal against the walls. The porcelain veneer should be so thin at the margins that it can easily be cracked by the instrument in the act of adaptation. The matrix is now removed and a second layer of Close body is applied the same as the first, and fused. If this layer was not too thick it will be found that the matrix now fits the cavity as it did when removed. If too much porcelain was used another adaptation will be necessary.

Having satisfactorily adapted the matrix to the tooth and this supported by the high-fusing body, the enamel can now be added without danger of contracting the matrix, provided not too much enamel is added at a fusing and only sufficient heat is used to fuse the same.

